# Identification of triple-negative breast cancer and androgen receptor expression based on histogram and texture analysis of dynamic contrast-enhanced MRI

**DOI:** 10.1186/s12880-023-01022-5

**Published:** 2023-06-01

**Authors:** Wen-juan Xu, Bing-jie Zheng, Jun Lu, Si-yun Liu, Hai-liang Li

**Affiliations:** 1 Department of Radiology, The Affiliated Cancer Hospital of Zhengzhou University & Henan Cancer Hospital, Zhengzhou, 450008 China; 2 GE healthcare (China), Beijing, 100176 China

**Keywords:** Breast cancer, Triple-negative breast cancer, Dynamic contrast-enhanced MRI, Texture analysis, Androgen receptor

## Abstract

**Background:**

Triple-negative breast cancer (TNBC) is highly malignant and has a poor prognosis due to the lack of effective therapeutic targets. Androgen receptor (AR) has been investigated as a possible therapeutic target. This study quantitatively assessed intratumor heterogeneity by histogram analysis of pharmacokinetic parameters and texture analysis on dynamic contrast-enhanced magnetic resonance imaging (DCE-MRI) to discriminate TNBC from non-triple-negative breast cancer (non-TNBC) and to identify AR expression in TNBC.

**Methods:**

This retrospective study included 99 patients with histopathologically proven breast cancer (TNBC: 36, non-TNBC: 63) who underwent breast DCE-MRI before surgery. The pharmacokinetic parameters of DCE-MRI (K^trans^, K_ep_ and V_e_) and their corresponding texture parameters were calculated. The independent t-test, or Mann-Whitney U-test was used to compare quantitative parameters between TNBC and non-TNBC groups, and AR-positive (AR+) and AR-negative (AR-) TNBC groups. The parameters with significant difference between two groups were further involved in logistic regression analysis to build a prediction model for TNBC. The ROC analysis was conducted on each independent parameter and the TNBC predicting model for evaluating the discrimination performance. The area under the ROC curve (AUC), sensitivity and specificity were derived.

**Results:**

The binary logistic regression analysis revealed that K_ep_Range_ (p = 0.032) and V_e_SumVariance_ (p = 0.005) were significantly higher in TNBC than in non-TNBC. The AUC of the combined model for identifying TNBC was 0.735 (p < 0.001) with a cut-off value of 0.268, and its sensitivity and specificity were 88.89% and 52.38%, respectively. The value of K_ep_Compactness2_ (p = 0.049), K_ep_SphericalDisproportion_ (p = 0.049), and V_e_GlcmEntropy_ (p = 0.008) were higher in AR + TNBC group than in AR-TNBC group.

**Conclusion:**

Histogram and texture analysis of breast lesions on DCE-MRI showed potential to identify TNBC, and the specific features can be possible predictors of AR expression, enhancing the ability to individualize the treatment of patients with TNBC.

## Introduction

Breast cancer is the most prevalent cancer in the world and has a high mortality rate [1]. According to the St Gallen International Expert Panel, invasive breast cancer is divided into five distinct subtypes based on microarray profiling: luminal A, luminal B, luminal-human epidermal growth factor receptor 2 (HER2), HER2-enriched, and triple negative (TN) [2]. Triple-negative breast cancer (TNBC) is defined by immunohistochemistry (IHC) as the lack of expression of estrogen receptor (ER), progesterone receptor (PR), and HER2 [3]. Compared with other subtypes of breast cancer, TNBC has an increased possibility of distant recurrence and death [4]. Currently, the treatment of breast cancer is largely guided by ER, PR and HER2 status: ER+/PR + patients require effective endocrine therapy; HER2 + patients require anti-HER2 targeted therapy; however, due to the lack of effective therapeutic targets for TNBC, there is no specific targeted therapy available. As a result, it has limited treatment options and is usually treated with cytotoxic therapy with poor clinical efficacy [5]. Therefore, it is important to distinguish TNBC from non-TNBC and to find new therapeutic targets for TNBC.

By adopting unsupervised clustering analyses with genomic data on cases of TNBC, six molecular subtypes of TNBC have been identified by Lehmann et al. [6], which have shown that TNBC is remarkably heterogeneous at the transcriptional level. Among them, the luminal androgen receptor (LAR) subtype characterized by androgen receptor (AR) expression is a subtype that deserves special attention.

AR has been reported as a prognostic biomarker that provides additional information and may be a viable therapeutic target for TNBC [7]. AR is highly expressed in 10–50% of TNBC [7]. According to some recent studies, AR has been shown to be a biomarker associated with poor prognosis in TNBC in terms of disease-free survival and overall survival [8]. The LAR subtype had a relatively lower proliferation rate, lower recurrence-free survival and similar distant metastasis-free survival compared with other subtypes [9]. Retrospective evaluation of patients undergoing neoadjuvant system therapy showed that LAR subtype is associated with lower pathologically complete response rates [10]. Furthermore, AR is under clinical investigation as a therapeutic target for TNBC [11, 12]. Therefore, identifying TNBC and judging the expression of AR in TNBC are of great significance in selecting treatment options and predicting treatment efficacy. If the presence of TNBC and the expression of AR in TNBC can be determined prior to surgery, it is possible to determine whether the patient is suitable for neoadjuvant chemotherapy and which regimen should be used. The identification of molecular subtypes and AR expression are mainly dependent on biopsy. However, due to the heterogeneity of the tumor, in up to 20% of patients, there is a difference in the receptor status between biopsy samples and postoperative samples [13]. Non-invasive prediction of molecular subtypes and AR expression based on MRI is a promising method to reflect the biology of the entire tumor and contribute to more accurate diagnosis and treatment. Therefore, numerous studies have attempted to use diffusion weighted imaging (DWI) [14, 15], DCE-MRI [16, 17], and other MRI sequences to differentiate molecular subtypes of breast cancer [18, 19]. However, there are few studies using MRI to distinguish whether AR is expressed in TNBC [20].

Among these sequences, DCE-MRI is considered the most sensitive method for detecting breast cancer. Some researchers looking at the image features on DCE-MRI found that the rim and persistent enhancement pattern of tumors were useful features for detecting TNBC [19]. Since the image characteristics of tumors are susceptible to the subjective influence of radiologists, some studies have attempted to identify TNBC with semi-quantitative parameters of DCE-MRI [21]. With the advancement of quantitative DCE-MRI based on pharmacokinetic models, there is an increasing interest in novel DCE-MRI-based biomarkers for identifying different molecular subtypes of breast cancer. Most of the relevant studies are histogram analysis that based on pharmacokinetic parameters [22]. The histogram is related to the grayscale frequency distribution of pixel intensities within the region of interest, and it provides a simple, visual representation of the statistical information contained in the image [23]. Histogram analysis based on pharmacokinetic parameters can reflect the blood perfusion of the tumor [24]. Researches in recent years have shown that texture analysis can reveal more information about heterogeneous tumor components [25]. Texture features quantify the gray-level changes within the image and contain deep information about the structural and organizational arrangement of the object and its connection with the surrounding environment.

Histogram and texture analysis of DCE-MRI can reveal more information about the heterogeneous tumor components [26]. We attempted to use histogram and texture features derived from multiple DCE-MRI parametric maps to noninvasively and preoperatively discriminate between TNBC and non-TNBC and identity whether AR is highly expressed in TNBC.

## Materials and methods

### Patient selection

As a retrospective study, we searched for 543 breast cancer patients who underwent breast DCE-MRI and subsequent treatment at our institution from June 2020 to August 2021. Inclusion criteria: (I) Patients with histologically confirmed invasive breast cancer and IHC findings including expression of AR, ER, PR, HER2 and Ki-67. (II) Patients underwent DCE-MRI within two weeks before surgery. (III) Patients with complete DCE-MRI data. (IV) Patients whose image quality and shooting conditions met the diagnostic criteria. Exclusion criteria: (I) Patients who received radiotherapy, neoadjuvant chemotherapy, or surgery before imaging. (II) Patients with incomplete DCE-MRI data or poor image quality. (III) Patients with other malignant tumors. (IV) Patients with incomplete pathological and immunohistochemical information. Finally, 99 female patients (mean age: 52.85 ± 10.15 years, range: 30–78 years) with 99 breast cancers (TNBC [n = 36], non-TNBC [n = 63]) were enrolled in this study. The study was conducted in accordance with the Declaration of Helsinki. The Life Science Ethics Committee of Zhengzhou University approved this retrospective analysis and waived the need for informed consent.

### DCE-MRI image acquisition

All examinations were performed on a 3.0 T Skyra device (Siemens Healthcare, Erlangen, Germany) using a 16-channel bilateral breast coil (Siemens Healthcare, Erlangen, Germany) with the patient positioned in the center of the magnet in the prone position. After routine fat-suppressed T2-weighted imaging, 3D gradient echo sequences with volumetric interpolated breath-hold examination at different flip angles (3°and 15°) were acquired for T1 mapping with the following parameters: repetition time (TR), 5.01 ms; echo time (TE), 2.26 ms; field of view (FOV), 340 × 340 mm^2^; slice thickness, 2.0 mm; matrix, 224 × 166; and total acquisition time (TA), 2 min 35 s. Next, DCE-MRI was performed using time-resolved angiography with interleaved stochastic trajectories sequence and the following parameters: TR, 4.18 ms; TE, 1.31 ms; FOV, 640 × 560 mm^2^; slice thickness, 2.0 mm; no gap; matrix, 320 × 249; flip angle, 12°; temporal resolution, 7.84 s/phase; and TA, 5 min 33 s. At the beginning of the fourth DCE-MRI frame acquisition, Gd-DTPA-BMA (0.2 mmol/kg; Omni-Scan, GE Healthcare, Ireland) was injected intravenously using a power injector at a flow rate of 2.5 mL/s, followed by a 20-mL saline-flush. T1 mapping and DCE-MRI were performed in the axial plane, and bilateral breast images were acquired.

### Postprocessing of MRI Data

**Fig. 1:**
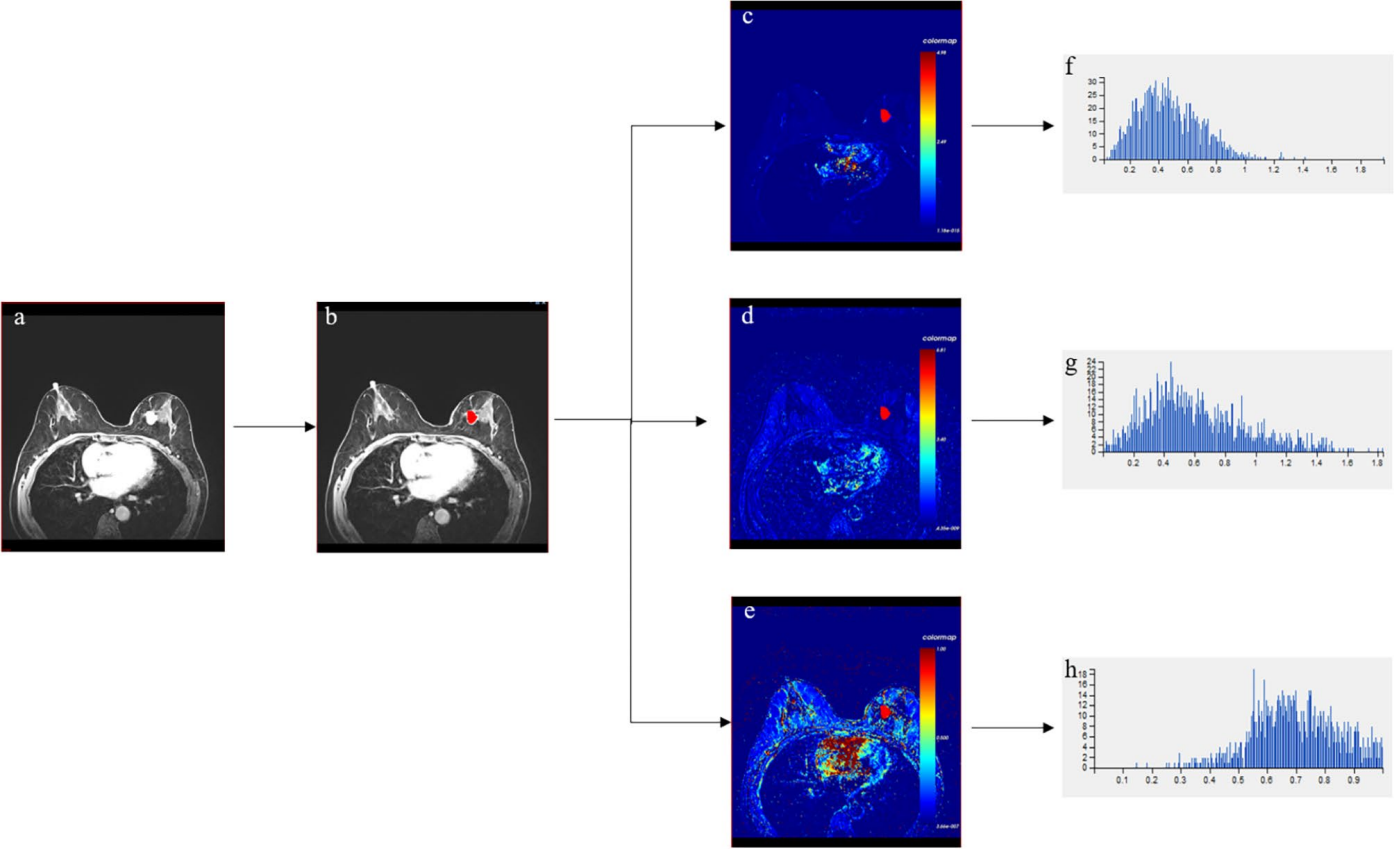
The workflow diagram shows the voxel analysis process for a tumor. (**a**) Post-contrast images. (**b**) ROI selection of tumor. (**c**) K^trans^ maps. (**d**) K_ep_ maps. (**e**) V_e_ maps. (**f-h**) Histograms of K^trans^ maps, K_ep_ maps and V_e_ maps, respectively. ROI, Region of interest

Raw DCE-MRI scan data were analyzed with a dedicated post-processing software (Omni-Kinetics; GE Healthcare, Milwaukee, WI). Two radiologists (BZ and WX) with 9 and 2 years of experience in the interpretation of breast MRI, who were blinded to the patients’ histopathological results, participated in this study. The enhancement kinetics were analyzed on the basis of the Extended Tofts Linear mode, and then the post-processing software automatically generated voxel-wise perfusion maps. Patient-specific artery input functions (AIFs) were measured, and tumor region of interest (ROI) was manually defined by radiologists on each axis of the lesion DCE-MRI sequence. Each layer of ROI included as much tumor tissue as possible, including any cystic, necrotic, or hemorrhagic components, for better assessment of tumor heterogeneity. To reduce the partial volume effect, the ROI was slightly smaller than the actual tumor size. Then, the workstation merged all ROIs into a volume of interest (VOI). The workflow was shown in Fig. 1. Finally, three pharmacokinetic parameters were extracted by the software, and the histograms and texture features corresponding to these parameters were also extracted. These parameters are K^trans^ (the transfer constant of the contrast agent from the plasma compartment into the extravascular extracellular space [EES], min^− 1^), K_ep_ (the rate constant of the escape of the contrast agent from the EES into the plasma compartment, min^− 1^), and V_e_ (the EES per unit volume of tissue, mL/100 mL of tissue, %).

To evaluate the interobserver agreement to verify the reliability and stability of these parameters, radiologist WX performed tumor measurements on all 99 patients and radiologist BZ performed tumor measurements on 50 patients randomly selected from the entire cohort. The parameters with interclass correlation coefficient (ICC) values greater than 0.75 for further analysis.

### Histopathologic assessment

The histopathological and immunohistochemical information was confirmed by surgical specimen. IHC analysis was used to determine the expression of ER, PR, AR, HER-2 and Ki-67. The expression of ER and PR was estimated by staining the cell nuclei; when the percentage of positive cells was higher than 1%, it was considered positive. HER2 status was determined by IHC or fluorescence in situ hybridization (FISH), an IHC score 3 + was defined as positive; when the IHC score was 2+, FISH was performed to assess gene amplification, and HER2 was considered positive if the ratio ≥ 2.0; while an IHC score 0 or 1 + was defined as negative. High expression of Ki-67 was defined as the presence of ≥ 14% positively stained nuclei. TNBC is defined as ER-negative, PR-negative and HER2-negative. The AR status was defined as positive if ≥ 1% of tumor cells showed positive staining.

### Statistical analysis

All data analyses were performed by SPSS (v26.0; Chicago, IL) and Medcalc(v19.6; Ostend, Belgium). Two-sided p < 0.05 was considered statistically significant. Normally distributed variables are expressed as the means ± standard deviations; abnormally distributed variables are expressed as the median with the first and third interquartile range. Respectively, the Kolmogorov-Smirnov test and the F-test were used to evaluate the normality and homogeneity of the variance of continuous variable. The independent t-test or Mann-Whitney U-test was used to compare quantitative parameters with histogram and texture features between two groups categorized based on molecular subtype (TNBC vs. non-TNBC) and AR status (AR + TNBC vs. AR-TNBC). The receiver operating characteristic curves (ROC) analysis was performed to evaluate the diagnostic performance of parameters between TNBC and non-TNBC and between AR + and AR-TNBC. Multivariate binary logistic regression (method: forward stepwise) was performed on the parameters that were significantly different between the TNBC and non-TNBC groups, and another ROC curve was drawn to determine the predictive accuracy of the model based on these parameters and to identify the best cut-off value for distinguishing TNBC.

## Results

### Patient clinicopathologic characteristics

In total, 99 patients with proven invasive ductal carcinoma were included in this study. The mean age of all the patients was 52.85 ± 10.15 years (age range: 30–78 years). Of the 99 patients, 36 patients (36.3%) were histologically confirmed TNBC and 63 patients (63.6%) were non-TNBC. No significant difference between the TNBC and non-TNBC groups were found in age, axillary lymph node status, and ductal carcinoma in situ (DCIS) status (p = 0.959, 0.243, 0.050, respectively). The features with statistically significant differences between the two groups were clinical T stage, histological grade, AR expression (p = 0.002, < 0.001, < 0.001, respectively). There are no significant differences in clinicopathologic characteristics between AR + and AR-TNBC groups. The tables of patient’s clinical pathologic characteristics were presented in Tables 1 and 2.

**Table 1:** Clinical and histopathologic characteristics of all people

Characteristics	non-TNBC (n = 63)	TNBC (n = 36)	p Value
Age	53.27 ± 10.49	52.11 ± 9.62	0.959
Histological grade			<0.001^*^
Low grade(grade 1 or 2)	52 (82.5%)	12 (33.3%)	
High grade(grade 3)	11 (17.5%)	24 (66.7%)	
Clinical T stage			0.002^*^
T1	45 (71.4%)	14 (38.9%)	
T2	18 (28.6%)	22 (61.1%)	
Axillary lymph node			0.243
Negative	42 (66.7%)	28 (77.8%)	
Positive	21 (33.3%)	8 (22.2%)	
DCIS status			0.050
Negative	46 (73.0%)	33 (91.7%)	
Positive	17 (27.0%)	3 (8.3%)	
AR			<0.001^*^
Negative	4 (6.3%)	20 (55.6%)	
Positive	59 (93.7%)	16 (44.4%)	

TNBC, Triple-negative breast cancer; non-TNBC, non-Triple-negative breast cancer; DCIS, Ductal carcinoma in situ; AR, Androgen receptor

^*^ indicates statistical significance at p < 0.05

**Table 2:** Clinical and histopathologic characteristics of people with triple-negative breast cancer

Characteristics	AR-TNBC (n = 20)	AR + TNBC (n = 16)	p Value
Age	49.5 ± 10.31	55.38 ± 7.78	0.068
Histological grade			0.729
Low grade (grade 1 or 2)	6 (30.0%)	6 (37.5%)	
High grade (grade 3)	14 (70.0%)	10 (62.5%)	
Clinical T stage			0.500
T1	7 (35.0%)	8 (50.0%)	
T2	13 (65.0%)	8 (50.0%)	
Axillary lymph node			0.422
negative	17 (85.0%)	11 (68.8%)	
Positive	3 (15.0%)	5 (31.2%)	
DCIS status			0.574
Negative	19 (95.0%)	14 (87.5%)	
Positive	1 (5.0%)	2 (12.5%)	

AR-TNBC, Androgen receptor-negative triple-negative breast cancer; AR + TNBC, Androgen receptor-positive triple-negative breast cancer; DCIS, Ductal carcinoma in situ

### TNBC versus non-TNBC

**Fig. 2:**
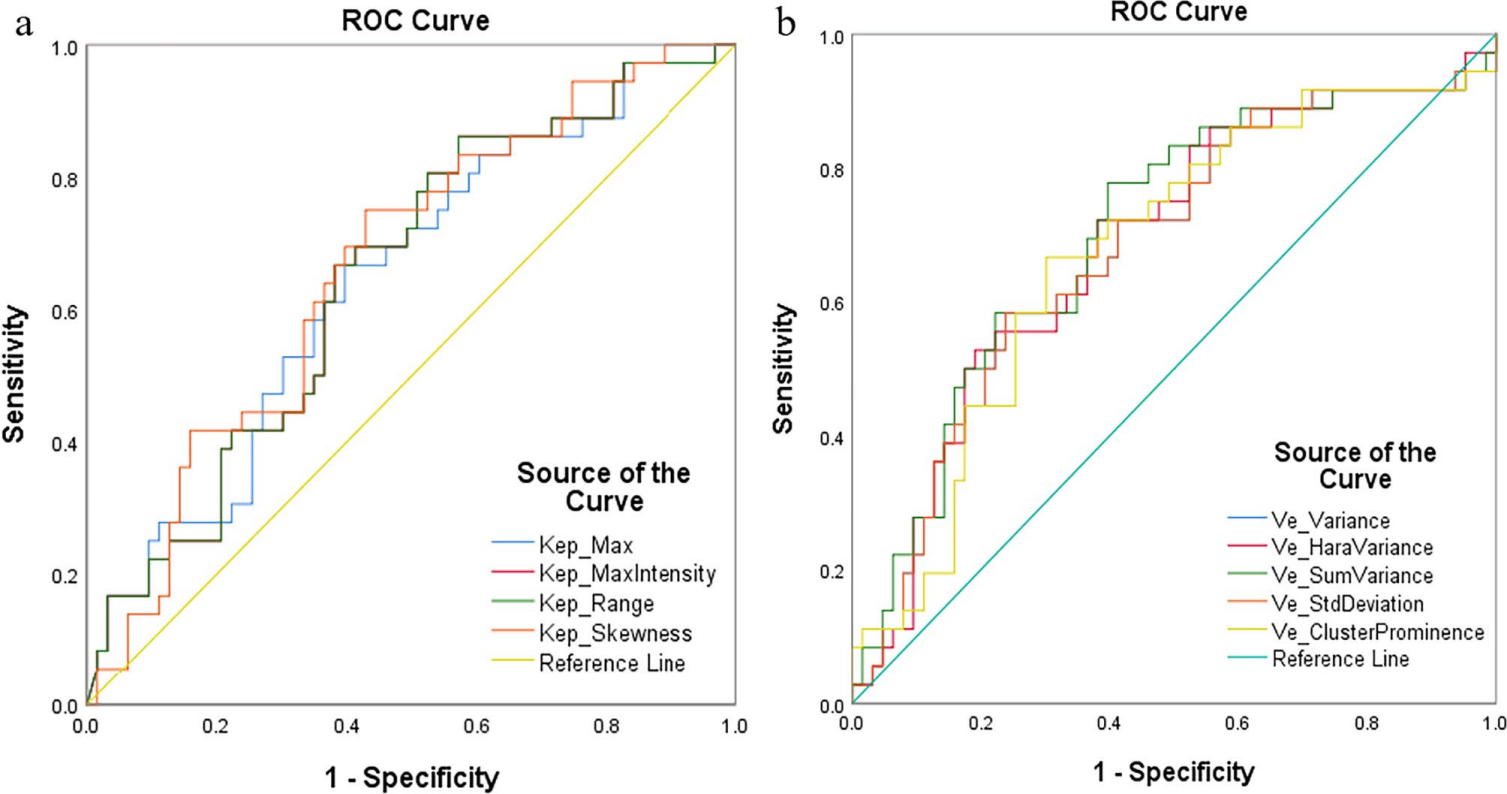
ROC curves for (**a**) K_ep_-related and (**b**) V_e_-related parameters that significantly differed between TNBC and non-TNBC. ROC, Receiver operating characteristic; TNBC, Triple-negative breast cancer; non-TNBC, non-Triple-negative breast cancer

After inter-observer agreement analysis, 261 histogram and texture features extracted from DCE-MRI were used for analysis. After the independent t-test and Mann-Whitney U-test, 9 parameters were found to be statistically different between the TNBC and non-TNBC groups, which were summarized in Table 3. The values of these ten parameters were all significantly higher in TNBC than in non-TNBC. The ROC curves and the AUC for all parameters were summarized in Table 4; Fig. 2. V_e_SumVariance_ showed the largest area under the ROC curve (AUC = 0.701, p = 0001), and AUCs of other parameters were near 0.700.

**Table 3:** Parameters that significantly different in TNBC from non-TNBC

Parameter	non-TNBC(n = 63)	TNBC(n = 36)	p Value
K_ep_Max_	3.075 ± 0.214	4.009 ± 0.316	0.018
K_ep_MaxIntensity_	2.809 (1.454, 3.621)	3.720 (2.545, 4.562)	0.014
K_ep_Range_	2.804 (1.454, 3.580)	3.719 (2.545, 4.562)	0.014
K_ep_Skewness_	1.051 ± 0.083	1.407 ± 0.098	0.009
V_e_Variance_	0.061 ± 0.005	0.084 ± 0.006	0.003
V_e_HaraVariance_	0.059 ± 0.005	0.083 ± 0.006	0.002
V_e_SumVariance_	0.043 ± 0.003	0.062 ± 0.005	0.001
V_e_StdDeviation_	0.236 (0.167, 0.283)	0.279 (0.236, 0.338)	0.003
V_e_ClusterProminence_(×10^7^)	3.594 (3.257, 9.856)	5.609 (4.687, 13.785)	0.004

The data in the table is represented by means ± standard deviations (normal distribution) or median (first and third quartiles) (skewed distribution). TNBC, Triple-negative breast cancer; non-TNBC, non-Triple-negative breast cancer

**Table 4:** Diagnostic value of histogram and texture parameters that can clearly distinguish TNBC from non-TNBC

Parameter	AUC	Standard Error	p Value	95% Confidence Interval	
				Lower Bound	Upper Bound
K_ep_Max_	0.643	0.057	0.018	0.531	0.755
K_ep_MaxIntensity_	0.649	0.056	0.014	0.539	0.760
K_ep_Range_	0.649	0.056	0.014	0.539	0.760
K_ep_Skewness_	0.664	0.055	0.007	0.555	0.773
V_e_Variance_	0.679	0.057	0.003	0.567	0.791
V_e_HaraVariance_	0.683	0.057	0.002	0.572	0.795
V_e_SumVariance_	0.701	0.056	0.001	0.592	0.811
V_e_StdDeviation_	0.679	0.057	0.003	0.567	0.791
V_e_ClusterProminence_	0.674	0.057	0.004	0.562	0.786

AUC, The area under the receiver operating characteristic curve

**Fig. 3:**
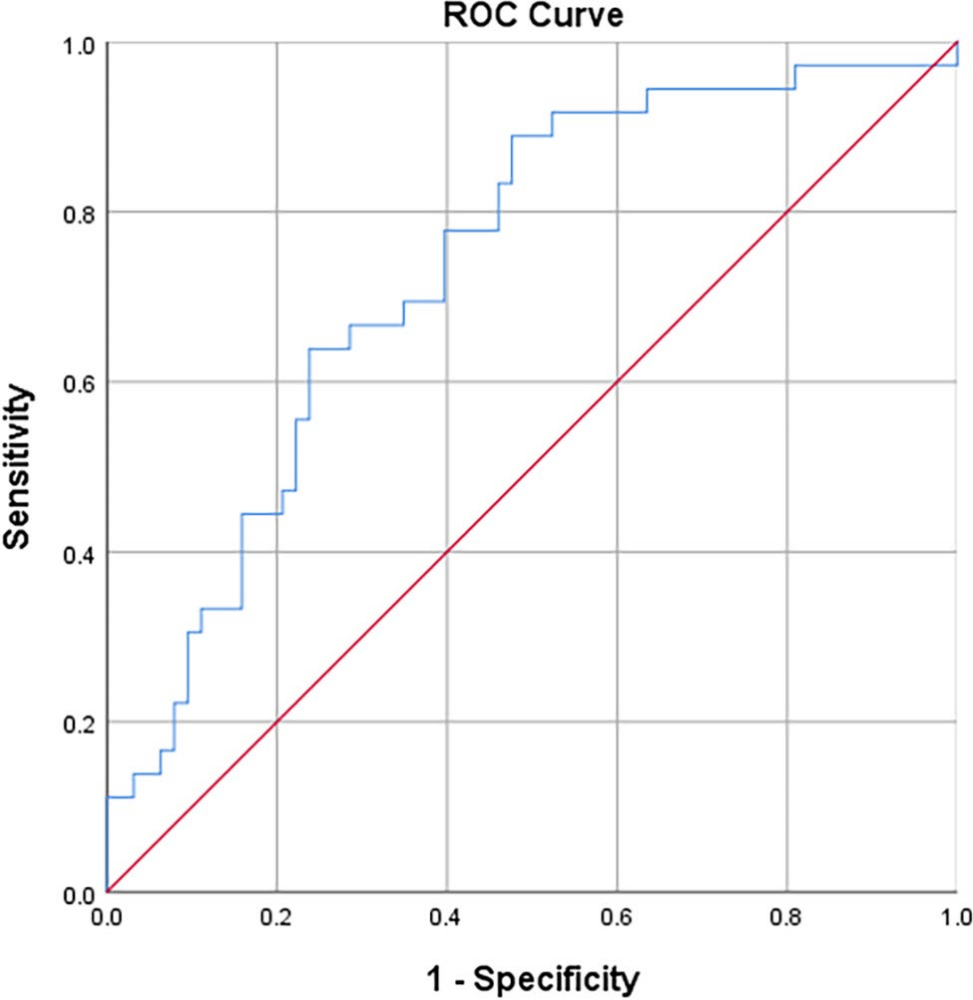
ROC curve for the combined model (blue line). ROC, Receiver operating characteristic

In the multivariate logistic regression analysis, only K_ep_Range_ (odds ratio = 1.326, p = 0.032) and V_e_SumVariance_ (odds ratio = 2.9 × 10^10^, p = 0.005) were associated with TNBC. The combined model was established based on these two parameters and the ROC curve was shown in Fig. 3. The AUC of the model for identifying TNBC was 0.735 (p < 0.001), the cutoff value, sensitivity and specificity were 0.268, 88.89% and 52.38%, respectively.

### AR + TNBC versus AR-TNBC

No histogram parameters were statistically different between the AR-TNBC and AR + TNBC groups. For the texture features, the values of K_ep_Compactness2_, K_ep_SphericalDisproportion_, and V_e_GlcmEntropy_ were significantly lower in the AR-TNBC group, which were summarized in Table 5. Table 6 showed the AUCs for these three parameters.

**Table 5:** Parameters that significantly different in AR-TNBC from AR + TNBC

Parameter	AR-TNBC	AR + TNBC	p Value
K_ep_Compactness2_	15.120 (13.497, 16.024)	16.845 (15.118, 18.040)	0.049
K_ep_SphericalDisproportion_	1.261 (1.172, 1.314)	1.354 (1.264, 1.422)	0.049
V_e_GlcmEntropy_	7.300 (5.685, 9.015)	8.587 (8.312, 10.445)	0.008

AR-TNBC, Androgen receptor-negative triple-negative breast cancer; AR + TNBC, Androgen receptor-positive triple-negative breast cancer

**Table 6:** Diagnostic value of histogram and texture parameters that can clearly distinguish AR + TNBC from AR-TNBC

Parameter	AUC	Standard Error	p Value	95% Confidence Interval	
				Lower Bound	Upper Bound
K_ep_Compactness2_	0.694	0.097	0.018	0.504	0.883
K_ep_SphericalDisproportion_	0.694	0.097	0.014	0.504	0.883
V_e_GlcmEntropy_	0.756	0.089	0.014	0.581	0.931

AUC, The area under the receiver operating characteristic curve

## Discussion

Our study sought to assess tumor heterogeneity by pharmacokinetic parameters with histogram and texture characteristics in preoperative DCE-MRI images of breast cancer patients. The current study aimed to distinguish TNBC from non-TNBC and to identify whether AR is expressed in TNBC, which would provide important imaging information for clinical treatment and prognosis prediction. We found that the K_ep_Range_ (p = 0.032) and V_e_SumVariance_ (p = 0.005) values of the TNBC group were higher than those of the non-TNBC group, and the AUC of their combined model was 0.735. The K_ep_Compactness2_, K_ep_SphericalDisproportion_, and V_e_GlcmEntropy_ values of the AR + TNBC group were higher than those of the AR-TNBC group, and their AUCs were all close to 0.700.

The incidence of TNBC in this study was approximately 36.3% of all breast cancers, which was higher than the 15–20% reported in previous study [3]. This may be attributed to the inclusion criteria of patients in this study and the differences in incidence rates in different regions. No difference in age and in the lymph node positivity rate was observed between TNBC and non-TNBC groups, although some studies have pointed out that TNBC is more likely to occur in younger women and has a higher lymph node positivity rate [4, 27]. The reason for this result may be that the number of patients was so small that the difference between the two groups did not reach statistical significance. Similar to previous reports [4, 28], TNBC in the present study was associated with higher histological grades.

Recently, several studies of TNBC MRI features have been reported. Morphologically, TNBC tends to present as a benign-like mass with a relatively circumscribed margin, which frequently shows internal high-signal intensity on T2-weighted images [29]. Under functional imaging, TNBC shows a higher apparent diffusion coefficient (ADC) on DWI because of a greater necrotic component [19]. On DCE-MRI, larger size, round/oval mass shape, smooth mass margin, rim and persistent enhancement pattern were useful features to detect TNBC [19, 30]. Through the quantitative analysis of DCE-MRI, several studies have observed significantly higher K_ep_ values in TNBC [31], which was similar to our results. Higher K_ep_ values are associated with increased vascular permeability, which may be attributed to the formation of new tumor vessels. However, the findings of Sung Hun Kim et al. were different and they believed that the K_ep_ value was independent of molecular subtype [32]. The reasons for this discrepancy may be related to differences in MRI protocols, study populations, ROI locations, and pharmacokinetic analysis software, and the substantial heterogeneity of breast cancers.

In our study, K_ep_Skewness_ represents the asymmetry of the probability distribution of the outflow rate of contrast agent between the interstitium and plasma. K_ep_Range_ represents the difference between the highest and lowest values of the outflow rate of contrast agent within VOI. The higher K_ep_Skewness_ and K_ep_Range_ of TNBC may be due to the fact that TNBC is densely packed with more aggressive components. Higher V_e_ values may imply poor tumor cellularity and an abundant tumor stroma, which includes fibroblasts, endothelial cells, and extracellular matrix components that can provide growth factors and then stimulate angiogenesis [32]. Variance represents the mean of the squared distances of each value in the image ROI from the mean of the values and standard deviation (StdDeviation) measures the amount of variation or dispersion from the mean of the values in the image ROI. Skewness and StdDeviation are biomarkers of tumor heterogeneity [23], our study found higher V_e_Variance_ and V_e_StdDeviation_ values for TNBC, which was in part consistent with the findings of Ken Nagasaka et al. [33], reflecting the high heterogeneity of TNBC. Texture features such as V_e_HaraVariance_, V_e_SumVariance_ and V_e_ClusterProminence_ were different in TNBC and non-TNBC groups in our study, which were different from the texture features found in the previous studies [34, 35], we speculate the differences might be due to study populations, ROI locations and postprocessing softwares. Our study further validated that texture features and histogram analysis can distinguish TNBC from non-TNBC.

Several studies have suggested that AR could be a potential therapeutic target for TNBC [7, 11]. Although gene expression profiling is still the gold standard for molecular typing of breast cancer [6], it is not common as it is time consuming and expensive. Currently, IHC does not routinely express AR in some hospitals, so some studies have explored the use of imaging to identify AR + TNBC. Previous studies reported that the heterogeneously dense breast composition, masses with calcifications and irregular shape on mammography, masses with irregular shape or spiculated margins on sonography, mammographic calcifications with or without a mass, and non-mass enhancement on MRI were useful features to detect AR + TNBC [20, 36]. This was the first study to predict AR expression in TNBC using DCE-MRI-based pharmacokinetic parameters containing histograms and texture features, and the results of this study suggest that DCE-MRI-based pharmacokinetic parameters containing texture features can be used to identify AR expression in TNBC.

Compared with previous studies that evaluated the histogram features of DCE-MRI in the characterization of TNBC [33], we outlined the region of interest layer by layer around the edge of the tumor and finally obtained a VOI and enrolled both histogram and texture features of DCE-MRI. Hence, the conclusion derived from our study might provide more quantitative information within the whole tumor volume.

There are some notable limitations of our study. First, this is a single-center study and should be verified through multi-center studies with different imaging equipment and protocols. Second, the number of patients in this study was relatively small. Although this was a cohort study of 99 patients, including more patients in the subgroup analysis would make the results more reliable. Third, this study is a retrospective study and further verification is required in a prospective study. Fourth, the manual ROI segmentation is time-consuming, so more convenient and accurate ROI segmentation methods need to be further explored in the future.

## Conclusions

The quantitative parameters of DCE-MRI and its corresponding histogram and texture parameters can help distinguish TNBC from non-TNBC and to identify whether AR is expressed in TNBC, which would provide important imaging information for clinical treatment and prognosis prediction.

## Data Availability

The datasets used and/or analysed during the current study are available from the corresponding author on reasonable request.

## Abbreviations

DCE-MRI: Dynamic contrast-enhanced magnetic resonance imaging
TNBC: Triple-negative breast cancer
non-TNBC: non-Triple-negative breast cancer
AR: Androgen receptor
AR+: AR-positive
AR-: AR-negative
ROC: Receiver operating characteristic
AUC: The area under the receiver operating characteristic curve
HER2: Human epidermal growth factor receptor 2
IHC: Immunohistochemistry
ER: Estrogen receptor
PR: Progesterone receptor
LAR: Luminal androgen receptor
DWI: Diffusion weighted imaging
TR: Repetition time
TE: Echo time
FOV: Field of view
TA: Total acquisition time
ROI: Region of interest
VOI: Volume of interest
EES: Extravascular extracellular space
ICC: Interclass correlation coefficient
FISH: Fluorescence in situ hybridization
DCIS: Ductal carcinoma in situ
ADC: Apparent diffusion coefficient
